# Supplementary material for: LncRNA modulates Hippo-YAP signaling to reprogram iron metabolism
Source: Nat Commun. 2023 Apr 20;14:2253. doi: 10.1038/s41467-023-37871-5 (PMC10119135; doi:10.1038/s41467-023-37871-5)
Supplement: Supplementary file 1 — Supplementary Information [file 41467_2023_37871_MOESM1_ESM.pdf]

LncRNA Modulates Hippo-YAP Signaling to Reprogram Iron Metabolism

Xin-yu He#[1,2,3], Xiao Fan#[1,2,3], Lei Qu[1,2,3], Xiang Wang[4], Li Jiang[5], Ling-jie Sang[1], Cheng-yu Shi[1], Siyi Lin[1], Jie-cheng Yang[1], Zuo-zhen Yang[1], Kai Lei[1], Jun-hong Li[1], Huai-qiang Ju[6], Qingfeng Yan[1], Jian Liu[7,8], Fudi Wang[5], Jianzhong Shao[1], Yan Xiong[9], Wenqi Wang*[10], and Aifu Lin*[,1,2,3,11,12]

#These authors have contributed equally.

*To whom correspondence should be addressed: linaifu@zju.edu.cn, wenqiw6@uci.edu，

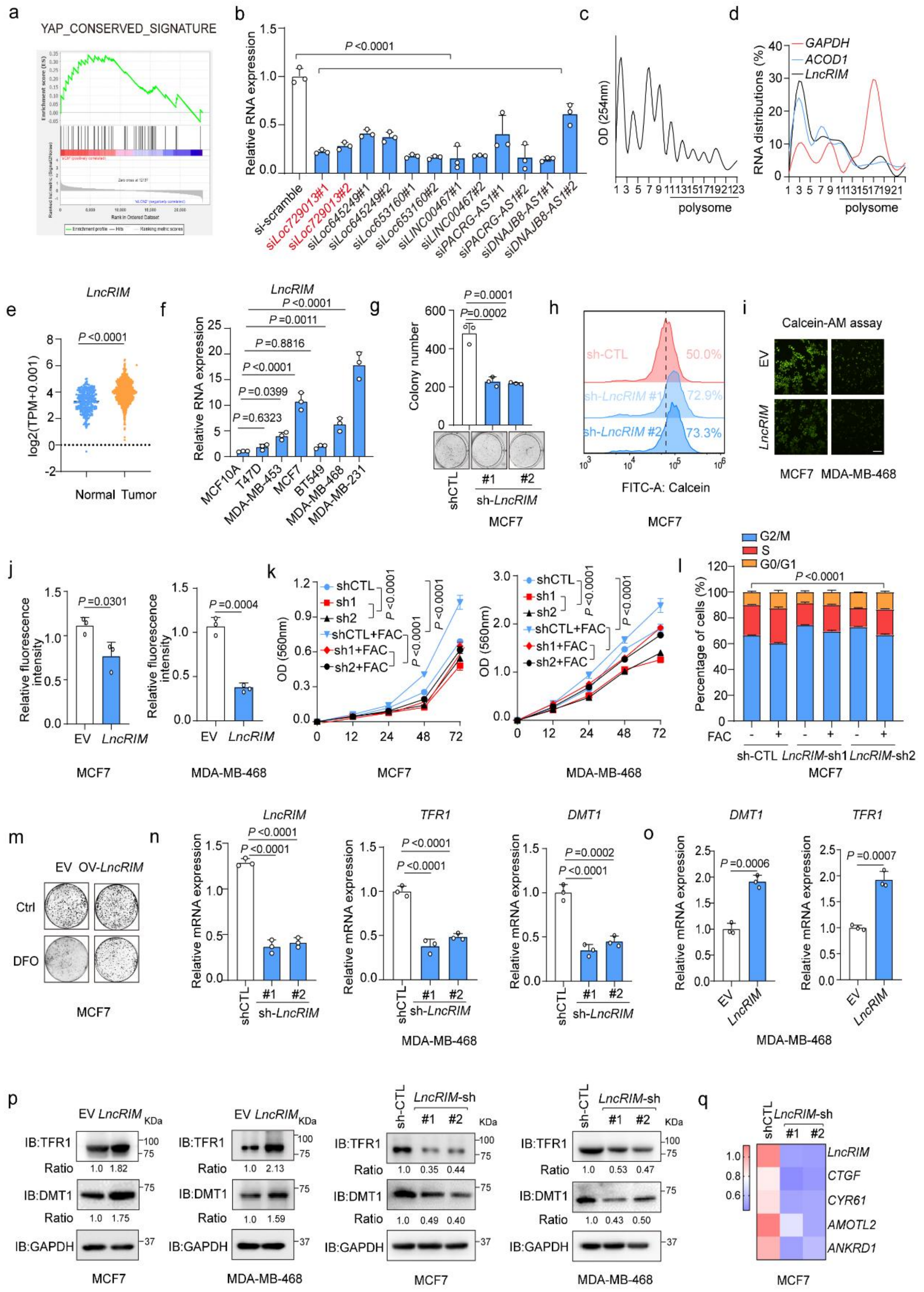

Supplementary Figure. 1 *LncRIM* regulates iron metabolism and breast cancer progression.

(**a**) Gene set enrichment analysis using the C6 canonical pathways Broad

MsigDB database on gene expression data compared control and the LCN2 knockdown MDA-MB-231 cell lines.(**b**)The knockdown efficiency of siRNA interference of the indicated candidates lncRNAs was examined by RT-qPCR. (mean ± SD, n = 3, One-way ANOVA analysis).(**c** and **d**) Polysome profiling of MCF-7 cells (**c**) and the amount of indicated transcript in each fraction was determined by RT-qPCR, and represented as percentage of all fractions (**d**). GAPDH was used as the canonical mRNA, and lncRNA *ACOD1* was used as the untranslated lncRNA.(**e**) Analysis of the *LncRIM* expression in normal (n=292)and tumor samples (n=1102)from TCGA database. (mean ± SD, two-sided Student's *t*-test) (**f**) RT-qPCR detection of *LncRIM* expression in different breast cancer cell lines. (mean ± SD, n = 3, One-way ANOVA analysis).(**g**) Colony formation assay of control and *LncRIM* knockdown MCF-7 cell lines.(mean ± SD, n = 3, One-way ANOVA analysis).(**h**) Calcein-AM and flow cytometry were performed to assess the cellular iron level of control and *LncRIM* knockdown MCF-7 cells.(**i** and **j**) The cellular iron level in EV and *LncRIM* overexpressed MCF-7 and MDA-MB-468 cells were assayed by Calcein-AM assay(**i**). The values was normalized to the control group (**j**). Scale bar 100 μm. (mean ± SD, n=3, two-sided Student's *t*-test).(**k**) Cell proliferation viability was assessed of EV and *LncRIM* overexpressed MCF-7 and MDA-MB-468 cells with or without FAC (200μM) stimulation. (mean ± SD, n = 3, Two-way ANOVA analysis).(**l**) Cell cycle analysis of control and *LncRIM* knockdown MCF-7 cells with or without FAC stimulation (200μM)

for 24hr. (mean ± SD, n = 3 ,Two-way ANOVA analysis).(**m**) Colony formation assay of EV and *LncRIM* overexpressed MCF-7 cells treated with or without DFO (100 μM) stimulation.(**n** and **o**) The DMT1 and TFR1 expression in control and *LncRIM* knockdown or *LncRIM* overexpressed MDA-MB-468 cells was examined by RT-qPCR. (mean ± SD, n = 3, One-way ANOVA analysis/two-sided Student's *t*-test).(**p**) Immunoblot detection of DMT1 and TFR1 in control and *LncRIM* knockdown or overexpressed MCF-7 and MDA-MB-468 cells.(**q**) RT-qPCR was performed to detect YAP targets expression of *LncRIM* knockdown MCF-7 cells.

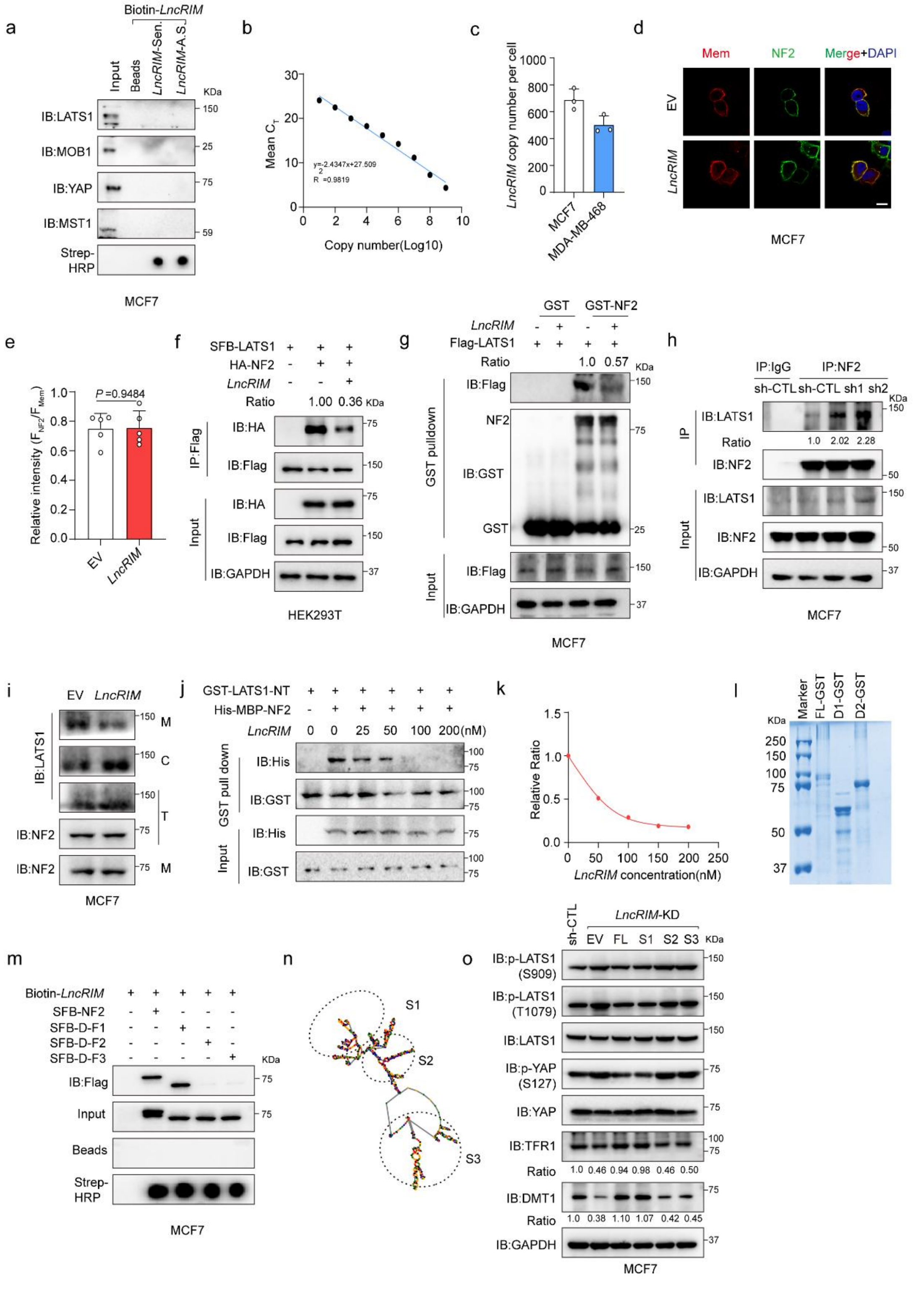

Supplementary Figure. 2 *LncRIM* interacts with NF2 to inactive LATS1 kinase.

(**a**) *In vitro*-transcribed biotinylated *LncRIM* sense (Sen.) or antisense (A.S.)

transcripts were incubated with MCF-7 cell lysates for the RNA pull-down assay, followed by immunoblot with indicated antibodies.(**b** and **c**) *LncRIM* copy number was determined in MCF-7 and MDA-MB-468 cell lines,n = 3.(**d** and **e**) Immunofluorescent staining of NF2 (Green) in control and *LncRIM* overexpressed MCF-7 cells. Cell membrane marker was stained with Red and the NF2 was detected with Alexa Fluor 488. Scale bar, 10 μm. The membrane location of NF2 (F_{NF2}/F_{Mem}) was analyzed with Image-J. (mean ± SD, n=5, two-sided Student's *t*-test).(**f**) Co-IP was performed to test the interaction between LAST1 and NF2 of HEK-293T cells expressing SFB-LATS1,HA-NF2 and *LncRIM*. (**g**) 20nmol GST-NF2 recombinant proteins were incubated with MCF-7 lysates expressing Flag-LATS1 and *LncRIM*. GST was used as the negative control. (**h**) An endogenous Co-IP was performed to assess NF2 and LATS1 interaction in control and *LncRIM* knockdown MCF-7 cells by NF2 antibodies. IgG was used as the negative control.(**i**) Subcellular fractionation assay was performed to assess LATS1 membrane association of control and *LncRIM* overexpressed MCF-7 cells. (M: membrane, C: cytoplasm, T: total).(**j** and **k**) 20nmol GST-LATS1-NT recombinant proteins and 20nmol His-MBP-NF2 recombinant proteins were incubated *in vitro* with different concentration of *in-vitro* transcribed biotinylated sense-*LncRIM.* GST-LATS1-NT was immunoprecipitated by GST beads.(l) Coomassie staining gel of the purified GST-NF2 and GST-NF2 mutants. The concentration was determined by the indicated BSA. (**m**) *In* vitro-transcribed biotinylated

LncRIM sense (Sen.) transcripts were incubated with MCF-7 cell lysates expressing different NF2 mutants. (**n**) The secondary structure of *LncRIM* was predicted by RNAFolder software and schematic illustration of *LncRIM* mutations. (**o**) Immunoblot detection of the DMT1, TFR1, LATS1, and YAP expression in control and *LncRIM* knockdown MCF-7 cells transfecting with full-length *LncRIM* or different truncations of *LncRIM.*

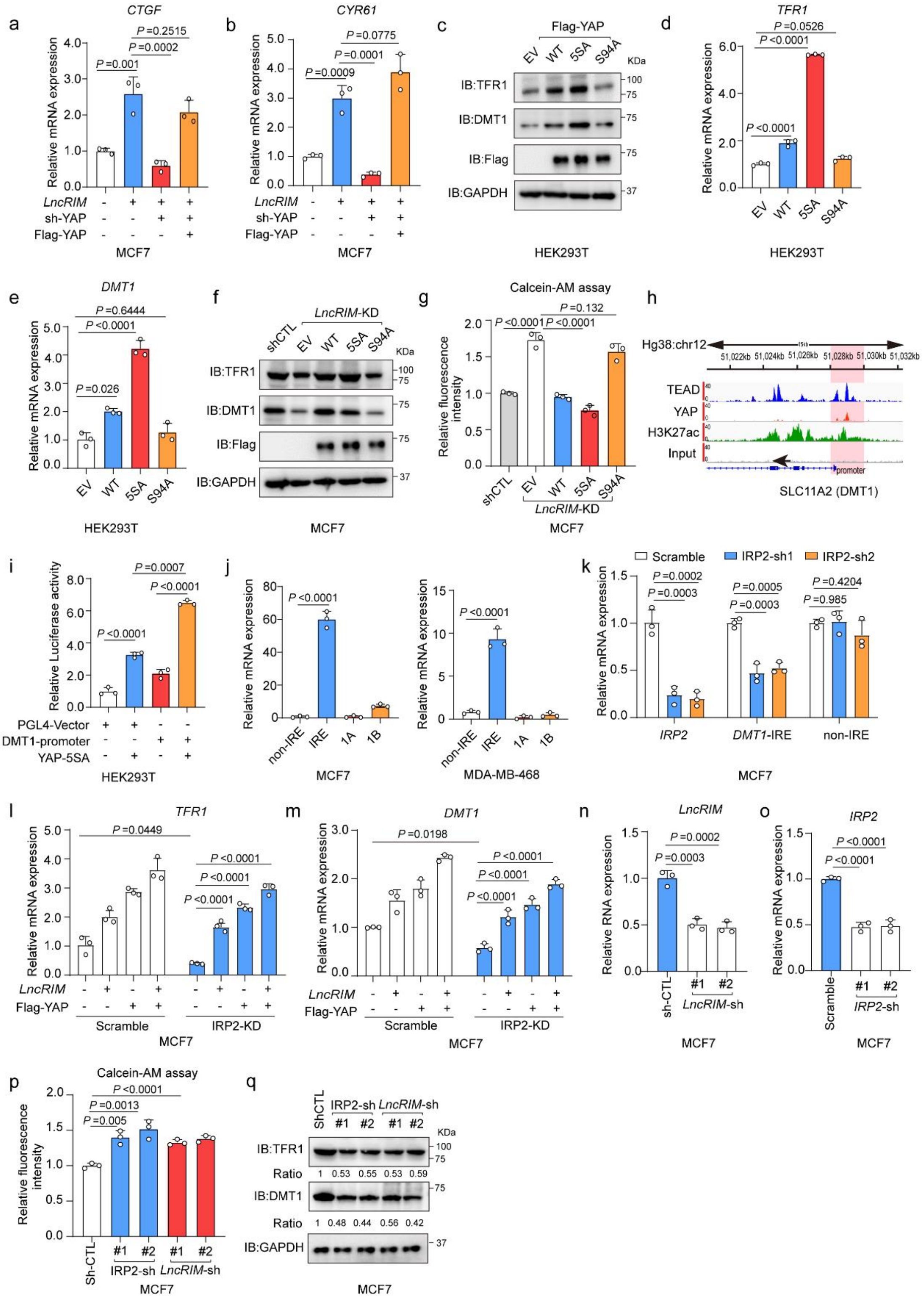

Supplementary Figure. 3 *LncRIM* modulates iron metabolism in a Hippo-YAP pathway-dependent manner.

(**a** and **b**) The expression of *CTGF* and *CYR61* of YAP knockdown MCF-7 cells

with or without re-expression of *LncRIM* was measured by RT-qPCR. (mean ± SD, n = 3, One-way ANOVA analysis).(**c-e**) Immunoblot (**c**) and RT-qPCR were performed to examine the expression of DMT1(**d**) and TFR1 (**e**) in MCF-7 cells stably expressing indicated YAP mutants. (mean ± SD, n = 3, One-way ANOVA analysis).(**f** and **g**) The DMT1 and TFR1 expression (**f**) , and the cellular iron level (**g**) of control and *LncRIM* knockdown MCF-7 cells transfecting with indicated YAP mutants was detected by immunoblot and Calcein-AM assay. (mean ± SD, n = 3, One-way ANOVA analysis).(**h**) ChIP-seq analysis of the YAP/TEAD binding elements in *DMT1* promoter region by using the data of GEO dataset (GSE107013).(**i**) Luciferase reporter assay was performed in MCF-7 cells with overexpression of YAP-5SA and DMT1-promoter. (mean ± SD, n = 3, One-way ANOVA analysis).(**j**) RT-qPCR detection of the four DMT1 isoforms expression in MCF-7 cells and MDA-MB-468 cells by using specific targets. (mean ± SD, n = 3, two-sided Student's *t*-test).(**k**) The expression of IRE-DMT1 and non-IRE DMT1 in control and IRP2 knockdown MCF-7 cells. (mean ± SD, n = 3, One-way ANOVA analysis).(**l** and **m**) The expression of DMT1 and TFR1 in control and IRP2 knockdown MCF-7 cells expressing Flag-YAP or *LncRIM* were measured by RT-qPCR. (mean ± SD, n = 3, Two-way ANOVA analysis).(**n** and **o**) RT-qPCR detection of knockdown efficiency of *LncRIM* (**n**) and IRP2 (**o**) in MCF-7 cells (mean ± SD, n = 3, One-way ANOVA analysis).(**p** and **q**) The cellular iron level (**p**), and the TFR1,DMT1 expression(**q**) in control and *LncRIM* knockdown or IRP2

knockdown MCF-7 cells were measured by calcein-AM assay and immunoblot.

(mean ± SD, n = 3, One-way ANOVA analysis).

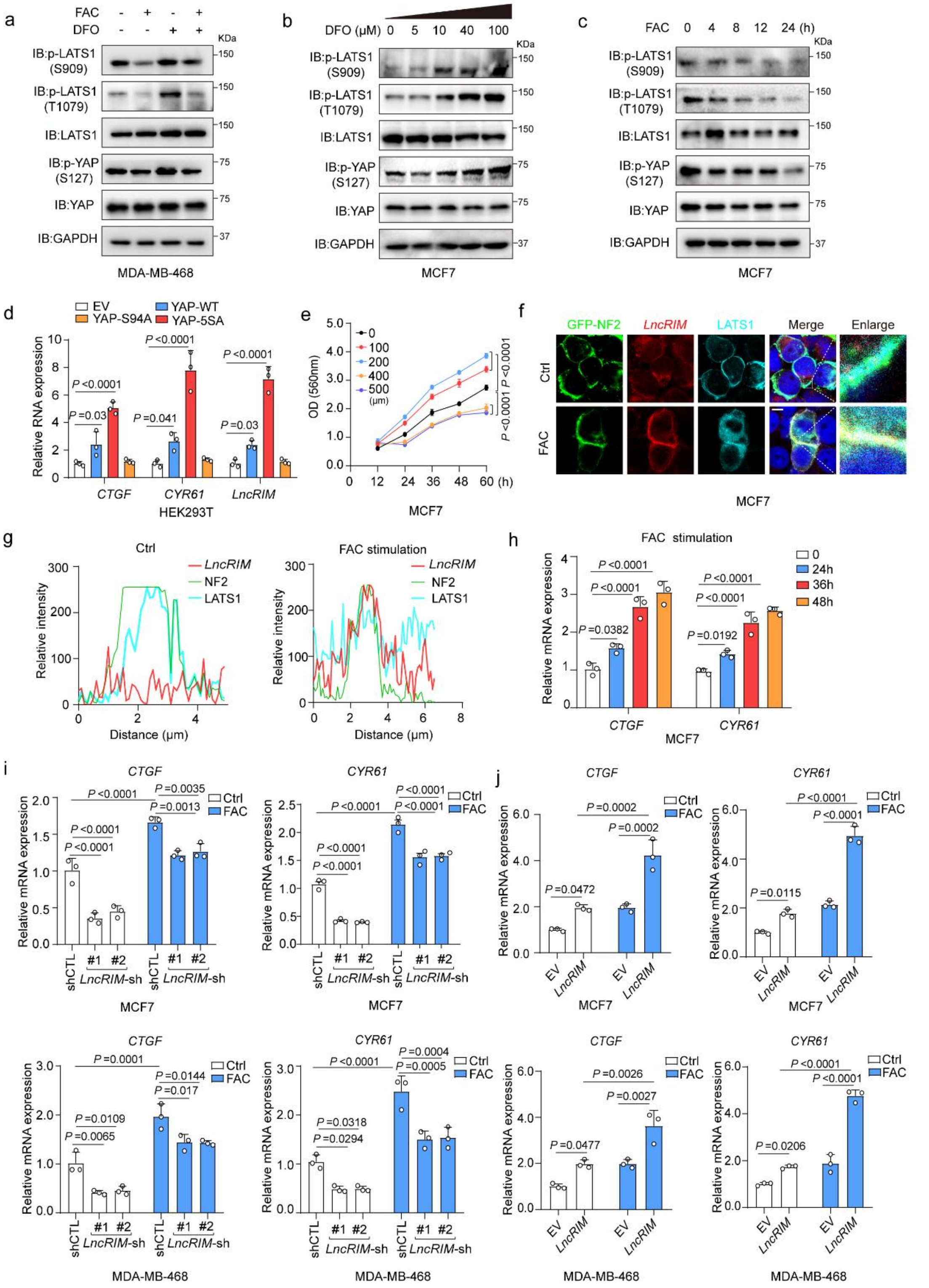

Supplementary Figure. 4 The iron-triggered *LncRIM*-NF2 feedback loop hyperactivates YAP .

(**a**) Immunoblot detection of p-YAP (S127), p-LATS1(S909, T1079) expression.

Serum-starved MDA-MB-468 cells are treated with FAC (200 μM) or DFO (100 μM) for 24 hr.(**b** and **c**) Immunoblot detection of p-YAP (S127), p-LATS1(S909, T1079) expression in serum-starved MCF-7 cells treated with different concentration of DFO for 24 hr (**b**), or treated with FAC (200 μM) for indicated time (**c**).(**d**) Actived YAP induces the transcription of *LncRIM* and YAP downstream targets. The expression of *LncRIM* and YAP downstream targets was examined by RT-qPCR in HEK-293T cells expressing indicated YAP mutants. (mean ± SD, n = 3, One-way ANOVA analysis).(**e**) Cell proliferation viability of MCF-7 cells treated with different concentration FAC was assessed by MTT assay. (mean ± SD, n = 3, Two-way ANOVA analysis).(**f** and **g**) Immunofluorescence staining was performed to assess the interaction between LATS1 and NF2 in MCF-7 cells expressing GFP-NF2(Green) with or without FAC stimulation (200μM) for 24hr. Line scan of the relative fluorescence intensity of the signal (**f**) is plotted to show the peak overlapping (**g**). The *LncRIM* probe was labeled with Cy3 (Red) and the LATS1 was detected with Alexa Fluor 647(Cyan). Scale bar, 20 μm.(**h**) The YAP downstream targets expression in MCF-7 cells treated with FAC (200μM) for different time were examined by RT-qPCR. (mean ± SD, n = 3, One-way ANOVA analysis).(**i** and **j**) RT-qPCR detection of the expression of *CTGF* and *CYR61* in control and *LncRIM* knockdown (**i**) or *LncRIM* overexpressed (**j**) MCF-7 and MDA-MB-468 cells with FAC stimulation (200μM) for 48hr (mean ± SD, n = 3, Two-way ANOVA analysis).

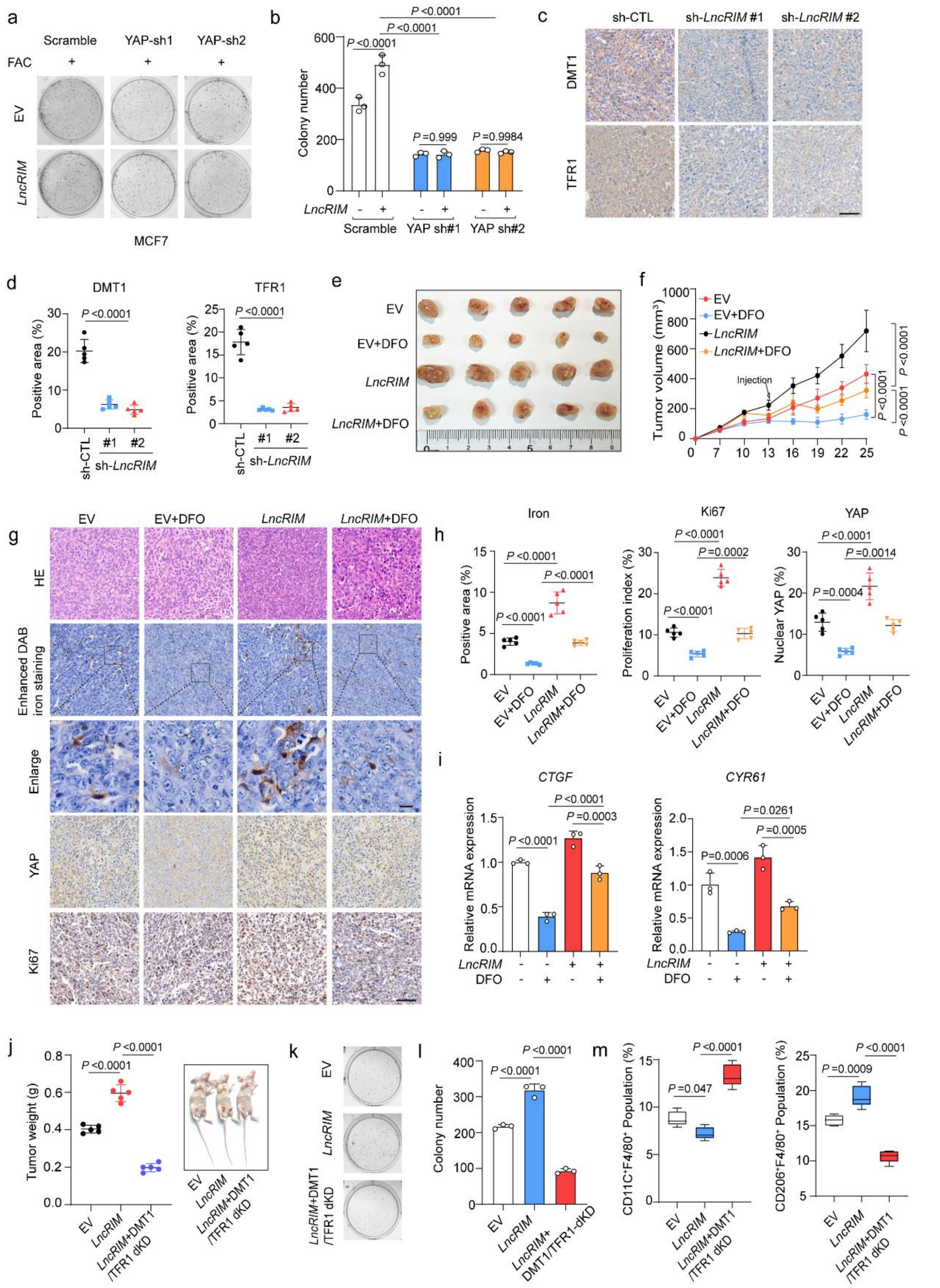

Supplementary Figure. 5 *LncRIM*-YAP axis-mediated iron metabolism promotes tumor progression.

(**a** and **b**) Colony formation assay of control and YAP knockdown MCF-7 cells

stimulated with FAC (200 μM). (mean ± SD, n = 3, Two-way ANOVA analysis).(**c** and **d**) IHC staining of DMT1 and TFR1 of randomly selected tumors from mice subcutaneously injected with the control or *LncRIM* knockdown MDA-MB-468 cells (**c**). Scale bar, 100 μm. The relative intensities of IHC were quantified by ImageJ(**d**). The data are presented as the mean ± SD of n = 5 mice per group, One-way ANOVA analysis.(**e** and **f**) Nude mice were injected with EV or *LncRIM* overexpressed MDA-MB-468 cells. After 13 days, two groups of nude mice were intraperitoneally injected with DFO (16 mg/kg) dissolved in 0.9% NaCl, once a day for 10 days. Tumor volumes were assessed (**f**). The data are presented as the mean ± SD of n = 5 mice per group, Two-way ANOVA analysis.(**g** and **h**) Representative IHC and iron staining of randomly selected tumors from mice subcutaneously injected with indicated MDA-MB-468 cell lines (**g**). Scale bar, 100 μm. The relative intensities were quantified by ImageJ (**h**). The data are presented as the mean ± SD of n = 5 mice per group, One-way ANOVA analysis.(**i**) RT–qPCR detection of YAP downstream genes of the indicated subcutaneous xenograft tumors. (mean ± SD, n = 3, One-way ANOVA analysis).(**j**) Tumor weight was detected of nude mice injected with control, *LncRIM* overexpressed, or double knockdown of DMT1/TFR1 with overexpression of *LncRIM* MDA-MB-468 cell lines. (mean ± SD,n = 5, One-way ANOVA analysis).(**k** and **l**) Colony formation assay of control, *LncRIM* overexpressed and double knockdown of DMT1/TFR1with overexpression of *LncRIM* MCF-7 cell lines.(mean ± SD, n =

3, One-way ANOVA analysis).(**m**) Flow cytometry analysis of M1 and M2-like tumor-associated macrophages from orthotopic injection tumor.(mean ± SD, n = 5, One-way ANOVA analysis). Data are presented as a box plot with box and whiskers. Bounds of box show the 25th and 75th percentiles, and the central lines in the box represent the median value. Whiskers show min to max value.Gating strategy is in the supplementary figure 6.

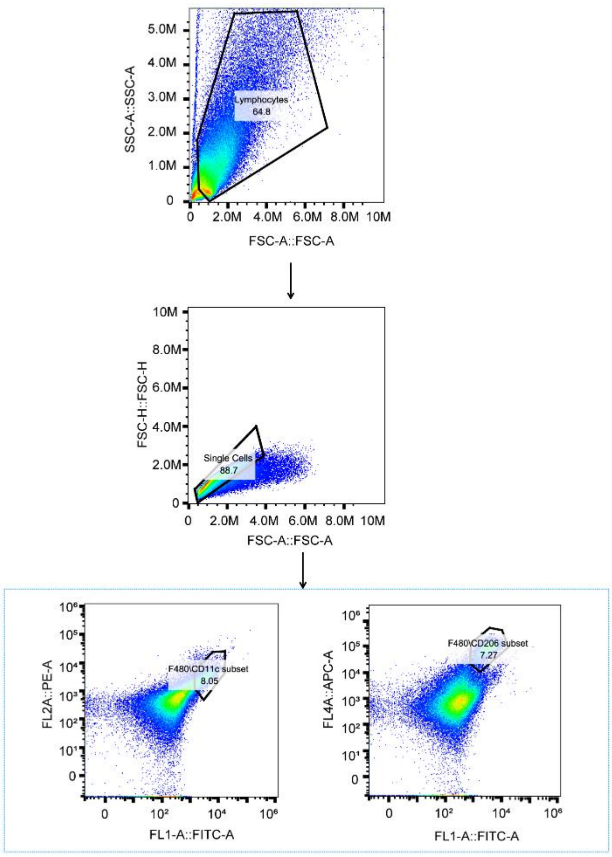

Supplementary Figure. 6 Gating strategy for detection of macrophages polarization.

Live cells in leukocyte fraction were gated via FSC and SSC, after exclusion of dead cells. Macrophages population was gated based on the surface expression of F4/80. In the blue dashed box : M1 macrophages population was gated based on the surface expression of CD11c. M2 macrophages population was gated based on the expression of CD206.

Table S1.Clinipathological Parameters of Tissue Microarrays Used in this Study. Related to Figure 1d

Breast cancer tissue array, including TNM and pathology grade, 69 cases/69 cores

Positon	Age	Sex	Organ	Pathological Diagnosis	Grade	Stage	TNM	Type
A1	60-70	F	Breast	Invasive ductal carcinoma	2	Ia	T1N0M0	Malignant
A2	40-50	F	Breast	Invasive ductal carcinoma	1	Ia	T1N0M0	Malignant
A3	50-60	F	Breast	Invasive ductal carcinoma	1	Ia	T1N0M0	Malignant
A5	60-70	F	Breast	Invasive ductal carcinoma	1	Ia	T1N0M0	Malignant
A6	30-40	F	Breast	Invasive ductal carcinoma	3	Ia	T1N0M0	Malignant
A5	70-80	F	Breast	Invasive ductal carcinoma	2	IIa	T2N0M0	Malignant
A6	70-80	F	Breast	Invasive ductal carcinoma	3	Ia	T1N0M0	Malignant
A8	30-40	F	Breast	Invasive ductal carcinoma	3	Ia	T1N0M0	Malignant
A9	40-50	F	Breast	Invasive ductal carcinoma	1	IIa	T2N0M0	Malignant
B1	50-60	F	Breast	Invasive ductal carcinoma	3	Ia	T1N0M0	Malignant
B2	60-70	F	Breast	Invasive ductal carcinoma	3	Ia	T1N0M0	Malignant
B3	20-30	F	Breast	Invasive ductal carcinoma	3	Ia	T1N0M0	Malignant
B4	40-50	F	Breast	Invasive ductal carcinoma	3	IIa	T2N0M0	Malignant
B5	80-90	F	Breast	Invasive ductal carcinoma	2	IIa	T2N0M0	Malignant
B6	50-60	F	Breast	Invasive ductal carcinoma	2	Ia	T1N0M0	Malignant
B7	70-80	F	Breast	Invasive ductal carcinoma	2	Ia	T1N0M0	Malignant
B8	60-70	F	Breast	Invasive ductal carcinoma	1	Ia	T1N0M0	Malignant
B9	60-70	F	Breast	Invasive ductal carcinoma	2	Ia	T1N0M0	Malignant
B10	70-80	F	Breast	Invasive ductal carcinoma	2	IIb	T2N1M0	Malignant
C1	50-60	F	Breast	Invasive ductal carcinoma	3	IIa	T2N0M0	Malignant
C2	80-90	F	Breast	Invasive ductal carcinoma	3	IIa	T2N0M0	Malignant
C3	50-60	F	Breast	Invasive ductal carcinoma	3	IIb	T2N1M0	Malignant
C4	70-80	F	Breast	Invasive ductal carcinoma	3	IIb	T2N1M0	Malignant
C5	50-60	F	Breast	Invasive ductal carcinoma	3	IIa	T1N1M0	Malignant
C6	40-50	F	Breast	Invasive ductal carcinoma	3	IIb	T2N1M0	Malignant
C7	70-80	F	Breast	Invasive ductal carcinoma	3	IIb	T2N1M0	Malignant
C8	80-90	F	Breast	Invasive ductal carcinoma	2	IIa	T2N0M0	Malignant
C9	50-60	F	Breast	Invasive ductal carcinoma	3	IIa	T2N0M0	Malignant
C10	70-80	F	Breast	Invasive ductal carcinoma	3	IIa	T1N1M0	Malignant
D1	50-60	F	Breast	Invasive ductal carcinoma	3	IIb	T2N1M0	Malignant
D2	40-50	F	Breast	Invasive ductal carcinoma	3	IIb	T2N1M0	Malignant
D3	60-70	F	Breast	Invasive ductal carcinoma	3	IIb	T2N1M0	Malignant
D4	50-60	F	Breast	Invasive ductal carcinoma	3	IIb	T3N0M0	Malignant
D5	30-40	F	Breast	Invasive ductal carcinoma	1	IIa	T2N0M0	Malignant
D6	40-50	F	Breast	Invasive ductal carcinoma	3	IIb	T2N1M0	Malignant
D7	70-80	F	Breast	Invasive ductal carcinoma	3	IIa	T1N1M0	Malignant
D8	60-70	F	Breast	Invasive ductal carcinoma	3	IIb	T2N1M0	Malignant
D9	50-60	F	Breast	Invasive ductal carcinoma	1	IIa	T2N0M0	Malignant
D10	70-80	F	Breast	Invasive ductal carcinoma	2	IIa	T2N0M0	Malignant
E1	40-50	F	Breast	Invasive ductal carcinoma	3	IIb	T2N1M0	Malignant
E2	60-70	F	Breast	Invasive ductal carcinoma	3	IIb	T2N1M0	Malignant
E3	60-70	F	Breast	Invasive ductal carcinoma	3	IIb	T2N1M0	Malignant
E4	60-70	F	Breast	Invasive ductal carcinoma	3	IIb	T2N1M0	Malignant
E5	40-50	F	Breast	Invasive ductal carcinoma	2	IIIa	T3N1M0	Malignant
E6	40-50	F	Breast	Invasive ductal carcinoma	3	IIIc	T3N3M0	Malignant
E7	30-40	F	Breast	Invasive ductal carcinoma	2	IIIc	T3N3M0	Malignant
E8	30-40	F	Breast	Invasive ductal carcinoma	3	IIb	T3N0M0	Malignant
E9	50-60	F	Breast	Invasive ductal carcinoma	3	IIIc	T3N3M0	Malignant
E10	50-60	F	Breast	Invasive ductal carcinoma	3	IIIa	T2N2M0	Malignant
F1	30-40	F	Breast	Invasive ductal carcinoma	3	IIIc	T2N3M0	Malignant
F2	50-60	F	Breast	Invasive ductal carcinoma	3	IIIa	T1N2M0	Malignant
F3	50-60	F	Breast	Invasive ductal carcinoma	3	IIIb	T4N4M0	Malignant
F6	60-70	F	Breast	Invasive ductal carcinoma	3	IIIc	T2N3M0	Malignant
F7	70-80	F	Breast	Invasive ductal carcinoma	3	IIIa	T3N1M0	Malignant
F8	40-50	F	Breast	Invasive ductal carcinoma	3	IIIb	T4N1M0	Malignant
F9	30-40	F	Breast	Invasive ductal carcinoma	3	IIIa	T3N1M0	Malignant
F10	60-70	F	Breast	Invasive ductal carcinoma	3	IIIc	T2N3M0	Malignant
G1	70-80	F	Breast	Invasive ductal carcinoma	1	Ia	T1N0M0	Malignant
G2	50-60	F	Breast	Invasive ductal carcinoma	1	Ia	T1N0M0	Malignant
G4	60-70	F	Breast	Invasive ductal carcinoma	1	IIa	T2N0M0	Malignant
G5	70-80	F	Breast	Invasive ductal carcinoma	1	Ia	T1N0M0	Malignant
G6	50-60	F	Breast	Invasive ductal carcinoma	1	Ia	T1N0M0	Malignant
G7	50-60	F	Breast	Invasive ductal carcinoma	1	Ia	T1N0M0	Malignant
H2	60-70	F	Breast	Invasive ductal carcinoma	2	IIa	T1N1M0	Malignant
H3	60-70	F	Breast	Invasive ductal carcinoma	1	IIa	T2N0M0	Malignant
H4	50-60	F	Breast	Invasive ductal carcinoma	3	IIa	T2N0M0	Malignant
H5	70-80	F	Breast	Invasive ductal carcinoma	3	IIa	T2N0M0	Malignant
H6	80-90	F	Breast	Invasive ductal carcinoma	3	IIa	T2N0M0	Malignant
H7	50-60	F	Breast	Invasive ductal carcinoma	3	IIa	T2N0M0	Malignant

Table S2. The correlation between clinicopathological parameters and *LncRIM* expression in Breast cancer.Related to Figure 1,6

	LncRIM expression		*P**
	Low, n(%)	High, n(%)	
**Age**			
＜45	15(45.5)	18(54.5)	0.5214
⩾45	34(52.3)	31(47.7)	
**Invasive depth**			
T1-T2	43(51.8)	40(48.2)	0.4
T3-T4	6(40.0)	9(60.0)	
**Lymph node metastasis**			
N=0	20(57.1)	15(42.9)	0.2918
N=1,2,3	29(46.0)	34(54.0)	
**TNM Stage**			
I-II	37(51.4)	35(48.6)	0.6472
III-IV	12(46.2)	14(53.8)	
**ER status**			
Negative	17(53.1)	15(48.4)	0.666
Positive	32(48.5)	34(51.4)	
**PR status**			
Negative	18(47.4)	20(52.6)	0.6784
Positive	31(51.7)	29(48.3)	
**HER2 status**			
Negative	19(51.6)	15(48.4)	0.6674
Positive	30(49.2)	34(50.8)	

**P* values determined by Chi-square test using SPSS 25.0. All statistical tests were two-sided. ER= Estrogen receptor; PR=Progesterone receptor.

Table S3. The correlation between clinicopathological parameters and *DMT1* expression in Breast cancer.Related to Figure 6

	DMT1 expression		*P**
	Low, n(%)	High, n(%)	
**Age**			
＜45	10(47.6)	11(52.4)	0.7994
⩾45	30(50.8)	29(49.2)	
**Invasive depth**			
T1-T2	36(55.4)	29(44.6)	0.045
T3-T4	4(26.7)	11(73.3)	
**Lymph node metastasis**			
N=0	15(48.4)	10(51.6)	0.2278
N=1,2,3	25(51.0)	30(49.0)	
**TNM Stage**			
I-II	30(55.6)	24(44.4)	0.1521
III-IV	10(37.5)	16(62.5)	
**ER status**			
Negative	10(37.0)	17(63.0)	0.098
Positive	30(56.6)	23(43.4)	
**PR status**			
Negative	11(39.3)	17(60.7)	0.1596
Positive	29(55.8)	23(44.2)	
**HER2 status**			
Negative	14(43.8)	18(56.2)	0.3613
Positive	26(54.2)	22(45.8)	

**P* values determined by Chi-square test using SPSS 25.0. All statistical tests were two-sided. ER= Estrogen receptor; PR=Progesterone receptor.

Table S4. The correlation between clinicopathological parameters and *TFR1* expression in Breast cancer. Related to Figure 6

	TFR1 expression		*P**
	Low, n(%)	High, n(%)	
**Age**			
＜45	8(38.1)	13(61.9)	0.2039
⩾45	32(54.2)	27(45.8)	
**Invasive depth**			
T1-T2	33(50.8)	32(49.2)	0.7745
T3-T4	7(46.7)	8(53.3)	
**Lymph node metastasis**			
N=0	11(44)	14(66)	0.4693
N=1,2,3	29(52.7)	26(47.3)	
**TNM Stage**			
I-II	28(519)	26(48.1)	0.6331
III-IV	12(46.2)	14(53.8)	
**ER status**			
Negative	8(29.6)	19(70.4)	0.8195
Positive	32(60.4)	21(39.6)	
**PR status**			
Negative	11(39.3)	17(60.7)	0.1596
Positive	29(55.8)	23(44.2)	
**HER2 status**			
Negative	13(42.4)	19(57.6)	0.1709
Positive	27(55.3)	21(44.7)	

**P* values determined by Chi-square test using SPSS 25.0. All statistical tests were two-sided. ER= Estrogen receptor; PR=Progesterone receptor.

Table S5. Sequences of Oligonucleotides for qPCR and RNA Interference.		
**Oligonucleotides Resource**	**Sequence**	**Application**
Loc729013 qF	CTCAAACTTCACTAGCCCCGT	RT-qPCR primer
Loc729013 qR	GAATGGGATGGGAGGACACA	RT-qPCR primer
GAPDH qF	CAGGGCTGCTTTTAACTCTGGTA	RT-qPCR primer
GAPDH qR	CATGGGTGGAATCATATTGGAAC	RT-qPCR primer
XIST qF	GACACAAGGCCAACGACCTA	RT-qPCR primer
XIST qR	TCGCTTGGGTCCTCTATCCA	RT-qPCR primer
SNHG6 qF	GCGTCAGCAAATCATGGACA	RT-qPCR primer
SNHG6 qR	TGTGCCACTTTCCTTGGTGG	RT-qPCR primer
ARRDC3-AS1 qF	CCAACCCTTCCCATCCAACT	RT-qPCR primer
ARRDC3-AS1 qR	ATCATGTTCTGTCGCCCCTC	RT-qPCR primer
LOC7299506 qF	CCTTGCTTTGCTGGGATGTG	RT-qPCR primer
LOC7299506 qR	AACTCAGTGCTGCCGATTGT	RT-qPCR primer
LOC84856 qF	AACTGCCCATACGGACCTAC	RT-qPCR primer
LOC84856 qR	GGTGAAAAGGCAGATCCCAAG	RT-qPCR primer
LEF1-AS1 qF	GGACTTCGCGGAAAAGGAGA	RT-qPCR primer
LEF1-AS qR	TTTTCACGGTCCCACGAGTT	RT-qPCR primer
C1orf213 qF	ATCCCCAGAACCTTGGCTAC	RT-qPCR primer
C1orf213 qR	CACTGGGAACAGGACACTCA	RT-qPCR primer
Clorf126 qF	CTGATTACCTCCCACGTGCC	RT-qPCR primer
Clorf126 qR	GGGCAAATTGCTTGCTGTCT	RT-qPCR primer
LOC728175 qF	GGGAAAAGGCCTCATCGACA	RT-qPCR primer
LOC728175 qR	GAATAGAGAGCCCGGAAGGC	RT-qPCR primer
PCOLCE-AS1 qF	TCACCTACCCAAGAAGGGGT	RT-qPCR primer
PCOLCE-AS1 qR	TGTGCCCAGCTAGAGTCAGA	RT-qPCR primer
PSMG3-AS1 qF	ACCTGGAAATTCAGCCGAGG	RT-qPCR primer
PSMG3-AS1 qR	TTGTGTGGTGGTGAGATCCG	RT-qPCR primer
TAPTF-AS1 qF	TTACAGGTCCACGAACTCGC	RT-qPCR primer
TAPTF-AS1 qR	GGCTGCCCTAACTTGGCATA	RT-qPCR primer
LOC439990 qF	ACCTGGAAATTCAGCCGAGG	RT-qPCR primer
LOC439990 qR	TTGTGTGGTGGTGAGATCCG	RT-qPCR primer
FLJ14107 qF	AAGTAGAAAACGCGCTCCCA	RT-qPCR primer
FLJ14107 qR	GACAACACCTGGTCTCCCTG	RT-qPCR primer
CLLU1 qF	TGCAGATACGTATGGCACCC	RT-qPCR primer
CLLU1 qR	ACACACATAAAGGGCAGCGA	RT-qPCR primer
RNASEH2B-AS1 qF	CGCTTTGAACTACCCTTGGC	RT-qPCR primer
RNASEH2B-AS1 qR	CTACTGGGTTGGAACCAGGG	RT-qPCR primer
CXORF28 qF	AGAGCCACACAGCAATGGAT	RT-qPCR primer
CXORF28 qR	TTGGTGGTGACGTTGGTTCA	RT-qPCR primer
DPYD-AS1 qF	GGCATATGCTCTTGCGATGC	RT-qPCR primer
DPYD-AS1 qR	GACACCCTTGGCTGTGATGA	RT-qPCR primer
SNHG1 qF	GCACGTTGGAACCGAAGAGA	RT-qPCR primer
SNHG1 qR	GCAGCTGAATTCCCCAGGATA	RT-qPCR primer
LOC728040 qF	ACAACTGCTAAGCTCTTTGGGA	RT-qPCR primer
LOC728040 qR	GCCAGGCTTAATTCGGCAAA	RT-qPCR primer
PAR-SN qF	AATCCAGTCAGTGTGCCTCA	RT-qPCR primer
PAR-SN qR	AGTGCAATACTACACCTGCCA	RT-qPCR primer
LOC643441 qF	CTGGGATCGAACAGTGGCTT	RT-qPCR primer
LOC643441 qR	GACAGGAAAGACTGGGTGGG	RT-qPCR primer
LOC728012 qF	AACCAGCGGGGTTACCTTTG	RT-qPCR primer
LOC728012 qR	TGCCCCGGTCTGATGTTTTC	RT-qPCR primer
ASAP1-1T1 qF	GACCCCTGCTTACCAATCCC	RT-qPCR primer
ASAP1-1T1 qR	GTCACCTCAGCTCCACGAAA	RT-qPCR primer
CFLAR-AS1 qF	AGAGACCTTATTTCGGCTGGC	RT-qPCR primer
CFLAR-AS1 qR	TAGTGCAGCACCCTTCATCTC	RT-qPCR primer
DNATB8-AS1 qF	ACACCAATGTGCGAATGCAG	RT-qPCR primer
DNATB8-AS1 qR	CTTCATGTGTGTGAGGCCGA	RT-qPCR primer
ZRANB2-AS1 qF	GTAACTGAGGCCCCATGAAGT	RT-qPCR primer
ZRANB2-AS1 qR	AACAAGATCACGGTCACCCG	RT-qPCR primer
LOC653160 qF	CCCGAATGTTCCCTGGATGT	RT-qPCR primer

LOC653160 qF	TTTTCCCGGGCCTAAGAAGG	RT-qPCR primer
Loc100128682 qF	GCCGTGACCCTCTTCAACTT	RT-qPCR primer
Loc100128682 qR	GAGCACTGAACAACACTGCG	RT-qPCR primer
Loc728724 qF	AGCCAAGAGGCTGGAGTTTC	RT-qPCR primer
Loc728724 qR	TGGTGGTGGTTGTATCAGGC	RT-qPCR primer
Clorf126 qF	ATCCCCAGAACCTTGGCTAC	RT-qPCR primer
Clorf126 qR	CACTGGGAACAGGACACTCA	RT-qPCR primer
Loc729013 qF	CTCAAACTTCACTAGCCCCGT	RT-qPCR primer
Loc729013 qR	GAATGGGATGGGAGGACACA	RT-qPCR primer
Loc645249 qF	AGGCCCCGCATTTTCAGATT	RT-qPCR primer
Loc645249 qR	GCTCTCAGCCTCGCCATAAA	RT-qPCR primer
LINC00467 qF	GCGTAGGCCGGACATTTCTA	RT-qPCR primer
LINC00467 qR	CCTGCCATGTTGGAAACTGC	RT-qPCR primer
PACRG-AS1 qF	CCTTTTGAGGCCACTTGCAC	RT-qPCR primer
PACRG-AS1 qR	TCCGTCTCCTCCGGATTTCT	RT-qPCR primer
TFR1 qF	GGACGCGCTAGTGTTCTTCT	RT-qPCR primer
TFR1 qR	CATCTACTTGCCGAGCCAGG	RT-qPCR primer
DMT1 qF	GCTCTCATACCCATCCTCACATT	RT-qPCR primer
DMT1 qR	TCCATTGGCAAAGTCACTCATT	RT-qPCR primer
TF qF	CTGGGAGCTTCTCAACCAGG	RT-qPCR primer
TF qR	TTGGCATTCATCCTTGGGGG	RT-qPCR primer
FTH1 qF	GGCAAAGTTCTTCAAAGCCA	RT-qPCR primer
FTH1 qR	CATCAACCGCCAGATCAAC	RT-qPCR primer
FTL qF	AACCATGAGCTCCCAGATTC	RT-qPCR primer
FTL qR	CGGTCGAAATAGAAGCCCAG	RT-qPCR primer
IRP1 qF	CCTGGAGTGTGGTAGGAACAC	RT-qPCR primer
IRP1 qR	GATCGAAAATGGTAAGCGCCC	RT-qPCR primer
IRP2 qF	AGCCTAAGAAGCTTCCCTGC	RT-qPCR primer
IRP2 qR	AGCCTAAGAAGCTTCCCTGC	RT-qPCR primer
FPN qF	TGGAAAGAAGGAAAAGAAAATCCC	RT-qPCR primer
FPN qR	GGTGCTTGTTAACAGGAGTGC	RT-qPCR primer
HEPH qF	CCAGACCTCTCTGGGATGTT	RT-qPCR primer
HEPH qR	TCTGTGCATGCTCATGGAGT	RT-qPCR primer
Hepcidin qF	TGACCAGTGGCTCTGTTTTCC	RT-qPCR primer
Hepcidin qR	GCAGCAGAAAATGCAGATGGG	RT-qPCR primer
CTGF qF	CCAATGACAACGCCTCCTG	RT-qPCR primer
CTGF qR	GAGCTTTCTGGCTGCACCA	RT-qPCR primer
CYR61 qF	AGCCTCGCATCCTATACAACC	RT-qPCR primer
CYR61 qR	GAGTGCCGCCTTGTGAAAGAA	RT-qPCR primer
AMOTL2 qF	AGCTTCAATGAGGGTCTGCT	RT-qPCR primer
AMOTL2 qR	TGAAGGACCTTGATCACTGC	RT-qPCR primer
DMT1-promoter-F	TTTGGGACCCACAGGTCATC	RT-qPCR primer
DMT1-promoter-R	GGGTTGGCTGCTCTCATTAT	RT-qPCR primer
ChIP-DMT1-F	TGATCAGTTTTCCGTGCTGC	RT-qPCR primer
ChIP-DMT1-R	TGGGAAGAAAATACATTGGCGG	RT-qPCR primer
ChIP-*LncRIM*-F	TTCGAAGGTGTTTCCCCGAA	RT-qPCR primer
ChIP-*LncRIM*-R	AAGGAGCATCTGTTCCCACC	RT-qPCR primer
DMT1-IRE-F	GAGCCAGTGTGTTTCTATGG	RT-qPCR primer
DMT1-IRE-R	CCTAAGCCTGATAGAGCTAG	RT-qPCR primer
non-IRE-F	GGGAAGGGTGTTCAAAACTG	RT-qPCR primer
non-IRE-R	CAATGCAGCACGGAAAACTG	RT-qPCR primer
1A-F	GGAGCTGGCATTGGGAAAGTC	RT-qPCR primer
1A-R	GGAGATCTTCTCATTAAAGTAAG	RT-qPCR primer
1B-F	GTTGCGGAGCTGGTAAGAATC	RT-qPCR primer
1B-R	GGAGATCTTCTCATTAAAGTAAG	RT-qPCR primer
siScramble	UUCUCCGAACGUGUCACGUTT	RNA interference
si*Loc729013* #1	GCCCUCUAAUGCCUGUAUA	RNA interference
si*Loc729013* #2	GAGACGAGCAUCUCACUAU	RNA interference
si*Loc653160* #1	GGACCUCUUUGUGGUCAAA	RNA interference
si*Loc653160* #2	GAGGCAAGAAAGGAACAGU	RNA interference
si*Loc645249* #1	CAGGGAAGAAGCACAAAUA	RNA interference
si*Loc645249* #2	GAGGGTTACACTGACAGAT	RNA interference

si*DNAJB8-AS1* #1	GACCUUGAGAAGAUUUAAA	RNA interference
si*DNAJB8-AS1* #2	GCACAAACCAGTCATTGAA	RNA interference
si*PACRG-AS1* #1	GGCUCAGGGAGAAUUUAAU	RNA interference
si*PACRG-AS1* #2	GCUGCAGCUUUCUGAUGAU	RNA interference
si*LINC00467* #1	GCUCUGUAAACCACAUAAU	RNA interference
si*LINC00467* #2	GCCAGACAGAUUCAAGUAU	RNA interference
shScramble	UUCUCCGAACGUGUCACGU	RNA interference
sh*Loc729013* #1	CTGCTTTCTGAAGTTATTATT	RNA interference
sh*Loc729013* #2	GGAAGCAATTGTTCTCAAACT	RNA interference
shNF2#1	GCAGGTGATTTGTCTTAATCA	RNA interference
shNF2#2	GGAGATCACACAACATTTATT	RNA interference
shYAP#1	GGTCAGAGATACTTCTTAAAT	RNA interference
shYAP#2	GACTCAGGATGGAGAAATTTA	RNA interference
shIRP2#1	GAGGATGAGAAGAAATTATTT	RNA interference
shIRP2#2	GGACCTAAATCAGAATCATAG	RNA interference
shIRP2#3	CCACCGGCAAGAACATTTACC	RNA interference
