# Supplementary material for: LncRNA modulates Hippo-YAP signaling to reprogram iron metabolism
Source: Nat Commun. 2023 Apr 20;14:2253. doi: 10.1038/s41467-023-37871-5 (PMC10119135; doi:10.1038/s41467-023-37871-5)
Supplement: Supplementary file 3 — Reporting Summary [file 41467_2023_37871_MOESM3_ESM.pdf]

nature portfolio

Corresponding author(s): Aifu Lin,Wenqi Wang

Last updated by author(s): Dec 20, 2022

Reporting Summary

Nature Portfolio wishes to improve the reproducibility of the work that we publish. This form provides structure for consistency and transparency in reporting. For further information on Nature Portfolio policies, see our Editorial Policies and the Editorial Policy Checklist.

Statistics

For all statistical analyses, confirm that the following items are present in the figure legend, table legend, main text, or Methods section.

n/a	Confirmed	
☐	☒	The exact sample size (*n*) for each experimental group/condition, given as a discrete number and unit of measurement
☐	☒	A statement on whether measurements were taken from distinct samples or whether the same sample was measured repeatedly
☐	☒	The statistical test(s) used AND whether they are one- or two-sided<br>*Only common tests should be described solely by name; describe more complex techniques in the Methods section.*
☒	☐	A description of all covariates tested
☐	☒	A description of any assumptions or corrections, such as tests of normality and adjustment for multiple comparisons
☐	☒	A full description of the statistical parameters including central tendency (e.g. means) or other basic estimates (e.g. regression coefficient) AND variation (e.g. standard deviation) or associated estimates of uncertainty (e.g. confidence intervals)
☐	☒	For null hypothesis testing, the test statistic (e.g. *F*, *t*, *r*) with confidence intervals, effect sizes, degrees of freedom and *P* value noted<br>*Give P values as exact values whenever suitable.*
☒	☐	For Bayesian analysis, information on the choice of priors and Markov chain Monte Carlo settings
☒	☐	For hierarchical and complex designs, identification of the appropriate level for tests and full reporting of outcomes
☐	☒	Estimates of effect sizes (e.g. Cohen's *d*, Pearson's *r*), indicating how they were calculated

Our web collection on statistics for biologists contains articles on many of the points above.

Software and code

Policy information about availability of computer code

Data collection	Commercial softwares equipped by CFX96 real-time PCR (CFX Manager, Bio-Rad), Tanon Image Analysis System (Tanon), ChemiDoc Touch Imaging System (Bio-Rad), Infinite M200 Pro (Tecan), FV3000 confocal microscope (Olympus), Super resolution Confocal Laser scanning microscope TCS SP8 STED (Leica), Nanodrop (Bio-Rad), SLIDEVIEW VS200 (Olympus), cytoFlex analyzer (Beckman Coulter).
Data analysis	Image Lab version 4.1 (Bio-Rad) was used to acquire Immunoblots and protein Coomassie staining gels. Acquired raw images were analyzed using FV31S-SW Viewer version 2.3.1 (Olympus), FV31S-DT version 2.3.1 (Olympus) and Leica Application Suite X version 3.3.0.16799 (Leica). The quantification of IHC staining and iron staining density was measured using Fiji Software version 2.3.0 (ImageJ, NIH) software. FlowJo Software version 7.6.4 (Biosciences) and CytExpert V2.3 (Beckman) was used to acquire flow cytometry. Statistical analysis was performed with GraphPad Prism version 8.0.2 and version 7.0.4 (GraphPad Software, Inc.). ClusterProflier (R package (4.1.4) was utilized to perform GSEA analysis.Bowtie2 (v2.3.5),SAMtools v1.9,MACS2 program (v2.2.4) were utilized to perform the ChIP-seq analysis.trim_galore (v.0.6.6), STAR (v.2.7.10b) , macs2 (v.2.2.7.1) and ChIPseeker(v.1.34.1) were utilized to analyze the binding motif of promoter.

For manuscripts utilizing custom algorithms or software that are central to the research but not yet described in published literature, software must be made available to editors and reviewers. We strongly encourage code deposition in a community repository (e.g. GitHub). See the Nature Portfolio guidelines for submitting code & software for further information.

Data

Policy information about availability of data

All manuscripts must include a data availability statement. This statement should provide the following information, where applicable:

- Accession codes, unique identifiers, or web links for publicly available datasets
- A description of any restrictions on data availability
- For clinical datasets or third party data, please ensure that the statement adheres to our policy

All the available datasets are included in the Data availability section.The Gene set enrichment analysis (GSEA) data in this study was available in the Broad MsigDB database (NCBI/GEO/GSE38369);The ChIP-seq analysis of YAP/TEAD in this study was available in the NCBI GEO database (NCBI/GEO/GSE107013); The analysis of LncRIM expression in tumor in this study was acquired using TCGA public database.

Human research participants

Policy information about studies involving human research participants and Sex and Gender in Research.

Reporting on sex and gender	In this study, all breast cancer patients were female, and the specific information was reported in Supplementary Table 1a.
Population characteristics	Information about breast cancer patients is included in Supplementary Table 1 including grade, age, gender, pathological diagnosis of both cancers patients.
Recruitment	All samples were collected from patients with informed consent, and all related procedures were performed with the approval of Institutional Review Board of The First People's Hospital of Huzhou. Patients were recruited with no perceived bias. All patients were not treated with adjuvant radiotherapy or chemotherapy before operation. All patients were provided with informed written consents for obtaining study specimens.
Ethics oversight	The internal review and ethics boards Institutional Review Board of The First People's Hospital of Huzhou.

Note that full information on the approval of the study protocol must also be provided in the manuscript.

Field-specific reporting

Please select the one below that is the best fit for your research. If you are not sure, read the appropriate sections before making your selection.

- [x] Life sciences
- [ ] Behavioural & social sciences
- [ ] Ecological, evolutionary & environmental sciences

For a reference copy of the document with all sections, see nature.com/documents/nr-reporting-summary-flat.pdf

Life sciences study design

All studies must disclose on these points even when the disclosure is negative.

Sample size	No statistical method was used to predetermine sample size. Sample size was chosen based on previous experience and standards in the field. All of the experiments were repeated at least three times. The sample size for in vitro (n>=3) and in vivo (n>=5) are typical in the field. We determined the sample size based on previous published studies in the field over the past years (Aifu Lin, et al., Nat Cell Biol. 2016; Aifu Lin, et al., Nat Cell Biol, 2017; Xin Zheng, et al. EMBO J, 2017; Lingjie Sang, et al., Mol Cell, 2018; Lingjie Sang, et al., Nat Metab, 2021).
Data exclusions	There are no data exclusions.
Replication	For each representative image/data, experiments were performed at least three times with similar results unless otherwise noted in the manuscript.
Randomization	Samples and organisms were randomly allocated to experimental groups. No specific randomization protocol has been used. Mice were ageand sex matched.
Blinding	No specific blinding was applied since all experiments were assigned into groups including relevalant controls and analysis was done objectively and without bias.

Reporting for specific materials, systems and methods

We require information from authors about some types of materials, experimental systems and methods used in many studies. Here, indicate whether each material, system or method listed is relevant to your study. If you are not sure if a list item applies to your research, read the appropriate section before selecting a response.

Materials & experimental systems

n/a	Involved in the study	
☐	☒	Antibodies
☐	☒	Eukaryotic cell lines
☒	☐	Palaeontology and archaeology
☐	☒	Animals and other organisms
☒	☐	Clinical data
☒	☐	Dual use research of concern

Methods

n/a	Involved in the study	
☒	☐	ChIP-seq
☐	☒	Flow cytometry
☒	☐	MRI-based neuroimaging

Antibodies

Antibodies used

anti-YAP (Cell Signaling Technology, #14074S)
anti-YAP (Proteintech,13584-1-AP)
anti-p-YAP (Ser127)(Cell Signaling Technology,#13008S)
anti-LAST1 (Cell Signaling Technology,#3477S)
anti-LATS1 (Proteintech,17049-1-AP)
anti-p-LATS1 (Ser909)(Cell Signaling Technology,#9157S)
anti-p-LATS1 (Thr1079)(Cell Signaling Technology,#8654S)
anti-Mob1 (Cell Signaling Technology,#13730S)
anti-NF2 (Cell Signaling Technology,#12888S)
anti-Mst1 (Cell Signaling Technology,#14946S)
anti-DMT1 (Proteintech,(20507-1-AP)
anti-TFR1 (Proteintech,10084-2-AP)
anti-TFR1 (ABclonal,A5865)
anti-FTH1 (ABclonal,A19544)
anti-IRP2 (Santa Cruz Biotechnology,sc-33682)
anti-GAPDH (Abmart,M20050)
anti-HA-tag (Abmart,M20003)
anti-DYKDDDDK-tag (Abmart,M20008)
anti-GST-tag (Abmart,M20007)
anti-His-tag (Abmart,M20001)
anti-TEAD4 (abcam,ab58310)
goat anti-rabbit IgG H&L (Alexa Fluor 488)(abcam,150077)
goat anti-rabbit IgG H&L (Alexa Fluor 647)(abcam,ab150083)
anti-CD11c-PE (eBioscience,#12-0114)
anti-CD206-APC (eBioscience,#17-2061)
anti-F4/80-FITC (Biolegend,#123116)
anti-ki67 (Cell Signaling Technology, #9449)
anti-CD31(ABclonal,A0378)

Validation

All antibodies used in this study are commercially available and all are validated by the vendors for the specific assays and species used; the validation data is available on the vendors website. As stated below, some antibodies are additionally validated in our study.
1) anti-YAP (Cell Signaling Technology, #14074S):
https://www.cellsignal.cn/products/primary-antibodies/yap-d8h1x-xp-rabbit-mab/14074
The manufacturer has validated this antibody for WB, IF and IHC in the species human and mouse.
2) anti-YAP (Proteintech,13584-1-AP)
https://www.ptgcn.com/products/YAP1-Antibody-13584-1-AP.htm
The manufacturer has validated this antibody for WB, IF and IHC in the species human and mouse.
3) anti-p-YAP (Ser127)(Cell Signaling Technology,#13008S)
https://www.cellsignal.cn/products/primary-antibodies/phospho-yap-ser127-d9w2i-rabbit-mab/13008
The manufacturer has validated this antibody for WB, IF and IHC in the species human and mouse.
4) anti-LAST1 (Cell Signaling Technology,#3477S)
https://www.cellsignal.cn/products/primary-antibodies/lats1-c66b5-rabbit-mab/3477
The manufacturer has validated this antibody for WB and IP in the species human and mouse.
5) anti-LATS1 (Proteintech,17049-1-AP)
https://www.ptgcn.com/products/LATS1-Antibody-17049-1-AP.htm
The manufacturer has validated this antibody for WB and IHC in the species human.
6) anti-p-LATS1 (Ser909)(Cell Signaling Technology,#9157S)
https://www.cellsignal.cn/products/primary-antibodies/phospho-lats1-ser909-antibody/9157
The manufacturer has validated this antibody for WB in the species human.
7) anti-p-LATS1 (Thr1079)(Cell Signaling Technology,#8654S)
https://www.cellsignal.cn/products/primary-antibodies/phospho-lats1-thr1079-d57d3-rabbit-mab/8654
The manufacturer has validated this antibody for WB in the species human and mouse.
8) anti-Mob1 (Cell Signaling Technology,#13730S)

https://www.cellsignal.cn/products/primary-antibodies/mob1-e1n9d-rabbit-mab/13730
The manufacturer has validated this antibody for WB and IP in the species human and mouse.
9)a nti-NF2 (Cell Signaling Technology,#12888S)
https://www.cellsignal.cn/products/primary-antibodies/merlin-d3s3w-rabbit-mab/12888
The manufacturer has validated this antibody for WB, IP and IF in the species human and mouse.
10) anti-Mst1 (Cell Signaling Technology,#14946S)
https://www.cellsignal.cn/products/primary-antibodies/mst1-d8b9q-rabbit-mab/14946
https://www.ptgcn.com/products/SLC11A2-Antibody-20507-1-AP.htm
11) anti-DMT1 (Proteintech,(20507-1-AP)
https://www.ptgcn.com/products/SLC11A2-Antibody-20507-1-AP.htm
The manufacturer has validated this antibody for WB and IHC in the species human and mouse.
12) anti-TFR1 (Proteintech,10084-2-AP)
https://www.ptgcn.com/products/TFRC-Antibody-10084-2-AP.htm
The manufacturer has validated this antibody for WB,IF,IP and IHC in the species human.
13) anti-TFR1 (ABclonal,A5865)
https://abclonal.com.cn/catalog/A5865
The manufacturer has validated this antibody for WB,IF,IP and IHC in the species human and mouse.
14) anti-FTH1 (ABclonal,A19544)
https://abclonal.com.cn/catalog/A19544
The manufacturer has validated this antibody for WB in the species human and mouse.
15) anti-IRP2 (Santa Cruz Biotechnology,sc-33682)
https://www.scbt.com/zh/p/irp-2-antibody-7h6
The manufacturer has validated this antibody for WB and IF in the species human.
16) anti-GAPDH (Abmart,M20050)
http://www.ab-mart.com.cn/page.aspx?node=%2059%20&id=%201247
The manufacturer has validated this antibody for WB in the species human.
17) anti-HA-tag (Abmart,M20003)
http://www.ab-mart.com.cn/page.aspx?node=%2059%20&id=%20963
The manufacturer has validated this antibody for WB in the species human.
18) anti--DYKDDDDK-tag (Abmart,M20008)
http://www.ab-mart.com.cn/page.aspx?node=%2059%20&id=%20968
The manufacturer has validated this antibody for WB in the species human.
19) anti-GST-tag (Abmart,M20007)
http://www.ab-mart.com.cn/page.aspx?node=%2059%20&id=%20967
The manufacturer has validated this antibody for WB in the species human.
20) anti-His-tag (Abmart,M20001)
http://www.ab-mart.com.cn/page.aspx?node=%2059%20&id=%20959
The manufacturer has validated this antibody for WB in the species human.
21) anti-TEAD4 (abcam,ab58310)
https://www.abcam.cn/products/primary-antibodies/tead4-antibody-5h3-ab58310.html
The manufacturer has validated this antibody for WB in the species human.
This antibody is validated for ChIP in PMID:32796823.
22) goat anti-rabbit IgG H&L (Alexa Fluor 488)(abcam,ab150077)
https://www.abcam.cn/products/secondary-antibodies/goat-rabbit-igg-hl-alexa-fluor-488-ab150077.html
The manufacturer has validated this antibody for IF in the species human.
23) goat anti-rabbit IgG H&L (Alexa Fluor 647)(abcam,ab150083)
https://www.abcam.cn/products/secondary-antibodies/goat-rabbit-igg-hl-alexa-fluor-647-preadsorbed-ab150083.html
The manufacturer has validated this antibody for IF in the species human and mouse.
24) anti-CD11c-PE (eBioscience,#12-0114)
https://www.thermofisher.cn/cn/zh/antibody/product/CD11c-Antibody-clone-N418-Monoclonal/12-0114-82
The manufacturer has validated this antibody for FC in the species mouse.
25) anti-CD206-APC (eBioscience,#17-2061)
https://www.thermofisher.cn/cn/zh/antibody/product/CD206-MMR-Antibody-clone-MR6F3-Monoclonal/17-2061-80
The manufacturer has validated this antibody for FC in the species mouse.
26) anti-F4/80-FITC (Biolegend,#123116)
https://www.biolegend.com/en-us/products/apc-anti-mouse-f4-80-antibody-4071
The manufacturer has validated this antibody for FC in the species]mouse.
27) anti-ki67 (Cell Signaling Technology, #9449)
https://www.cellsignal.cn/products/primary-antibodies/ki-67-8d5-mouse-mab/9449
The manufacturer has validated this antibody for IHC and WB in the species Human.
28) anti-CD31(ABclonal,A0378)
https://abclonal.com.cn/catalog/A0378
The manufacturer has validated this antibody for IHC and WB in the species Human and mouse.

Eukaryotic cell lines

Policy information about cell lines and Sex and Gender in Research

Cell line source(s)	The human breast cancer cell lines MDA-MB-468 (HTB-132; RRID: CVCL_0419), MCF7 (HTB-22; RRID: CVCL_0031), MDA-MB-453 (HTB-131; RRID:CVCL_0418), MDA-MB-231(CRM-HTB-26; RRID: CVCL_0062), BT549(HTB-122 , RRID: CVCL1092), T47D (CRL-2865 , RRID: CVCL_0553), the human epithelial cell MCF10A(CRL-10317, RRID: CVCL_0598), and the human embryonic kidney cell line HEK293T(CRL-3216; RRID: CVCL_0063) were purchased from National Collection of Authenticated Cell Cultures (China).
Authentication	All cell lines were authenticated based on STR fingerprinting before use.
Mycoplasma contamination	All cell lines were tested negative for mycoplasma contamination. Stated in Methods section.
Commonly misidentified lines (See ICLAC register)	No commonly misidentified cell lines were used.

Animals and other research organisms

Policy information about studies involving animals; ARRIVE guidelines recommended for reporting animal research, and Sex and Gender in Research

Laboratory animals	All animal experiments were performed by a protocol approved by the Institutional Animal Care and Use Committee (IACUC), and the mice had a maximum tumor size/burden of less than 15 mm. The care of experimental animals was by appropriate guidelines and approved by the Laboratory Animal Committee of Zhejiang University (ZJU20210028). Female nude mice (BALB/c strain; 4-5 weeks old) were purchased from the Shanghai Laboratory Animals Center and used in the xenograft mouse model assay. Animals were housed in a pathogen-free barrier environment (approximately 20 °C with 40% humidity and a 12-hr dark/light cycle) throughout the study. Mice were fed a normal chow diet and water with ad libitum feeding. Control and experimental animals were bred separately.
Wild animals	This study did not involve the wild animals.
Reporting on sex	Since this study focused on breast cancer models, only female mice were used as study subjects.
Field-collected samples	This study did not include field-collected samples.
Ethics oversight	Animals were housed in a pathogen-free barrier environment (around 20°C with 40% humidity and 12-h dark/light cycle) throughout the study, and experimental protocols were approved by the Animal Care and Use Committee of Zhejiang University School of Medicine.

Note that full information on the approval of the study protocol must also be provided in the manuscript.

Flow Cytometry

Plots

Confirm that:

- [x] The axis labels state the marker and fluorochrome used (e.g. CD4-FITC).
- [x] The axis scales are clearly visible. Include numbers along axes only for bottom left plot of group (a 'group' is an analysis of identical markers).
- [x] All plots are contour plots with outliers or pseudocolor plots.
- [x] A numerical value for number of cells or percentage (with statistics) is provided.

Methodology

Sample preparation	MDA-MB-468 cells were subsequently injected orthotopically into nude mice. After three weeks, mice were sacrificed, and the tumors were dissected. Tumor tissues were minced and excised into small pieces followed by incubation in DMEM containing 1 mg/mL collagenase IV (Sigma, C4-28-100MG) and 10–3U/L DNase I (Invitrogen, EN0521) for 0.5-1h. After lysed, single-cell suspensions were stained with fluorochrome-conjugated antibodies.
Instrument	CytoFlex analyzer (Beckman Coulter)
Software	FlowJo X, CytExpert v2.3 and GraphPad Prism 8.0
Cell population abundance	Moderate
Gating strategy	In our experiment, FITC conjugated-F4/80 antibody was used to gate the living macrophages cells, APC conjugated-CD206 antibody was used to circle the M2 macrophage cells, and PE conjugated-CD11c antibody was used to gate the M1 macrophage cells.

- [x] Tick this box to confirm that a figure exemplifying the gating strategy is provided in the Supplementary Information.
